# Memory advantage for untrustworthy faces: Replication across lab- and web-based studies

**DOI:** 10.1371/journal.pone.0264034

**Published:** 2022-02-17

**Authors:** Manon Giraudier, Carlos Ventura-Bort, Julia Wendt, Alexander Lischke, Mathias Weymar

**Affiliations:** 1 Department of Biological Psychology and Affective Science, Faculty of Human Sciences, University of Potsdam, Potsdam, Germany; 2 Department of Psychology, Medical School Hamburg, Hamburg, Germany; 3 Institute of Clinical Psychology and Psychotherapy, Medical School Hamburg, Hamburg, Germany; 4 Faculty of Health Sciences Brandenburg, University of Potsdam, Potsdam, Germany; University Hospitals Tubingen: Universitatsklinikum Tubingen, GERMANY

## Abstract

The Covid-19 pandemic imposed new constraints on empirical research and forced researchers to transfer from traditional laboratory research to the online environment. This study tested the validity of a web-based episodic memory paradigm by comparing participants’ memory performance for trustworthy and untrustworthy facial stimuli in a supervised laboratory setting and an unsupervised web setting. Consistent with previous results, we observed enhanced episodic memory for untrustworthy compared to trustworthy faces. Most importantly, this memory bias was comparable in the online and the laboratory experiment, suggesting that web-based procedures are a promising tool for memory research.

## Introduction

The Covid-19 pandemic has caused significant disruptions to all aspects of life and work. Due to the need to reduce social contact, the work environment has suffered a radical transformation. Scientific research has not been an exception and many researchers, particularly in the field of psychology and neuroscience, have been forced to transfer lab-based behavioral research to the online environment [1, 2]. Although web-based research has some inherent limitations due to lack of experimental control and potential technical challenges (e.g., variations in internet speed and display settings), as well as unknown participant behavior (through anonymous and unsupervised participation), it also has some advantages over traditional laboratory settings: it allows the recruitment of large and diverse samples of participants in terms of age, gender, origin, culture and social status, minimizes organizational issues such as scheduling conflicts and time constraints, eliminates potential experimenter effects, and reduces costs related to laboratory space, personnel hours, equipment, and administration [3–10].

Recent studies also indicated that online experiments show comparable results to those conducted in the laboratory environment [11–18]. For instance, Crump and colleagues [14] examined the similarities between lab- and web-based settings in a series of behavioral experiments, including Stroop, Switching, Flanker, and Simon tasks. The authors observed that the web-based environment replicated the experimental standard effects found in a traditional laboratory setting [14]. However, the authors also addressed disparities between lab-based and web-based research, possibly due to timing differences of participants’ web browsers and other technical challenges. While web-based experimental procedures allow for efficient data collection with results comparable to those of laboratory experiments, there is still reason for caution, and the validity of web settings needs to be further empirically determined.

Therefore, the primary goal of the present study was to test the reliability of web-based tests in experimental psychology and related domains further. We focused on an episodic memory task (recognition memory), which has been conducted both online and in a laboratory setting. Deficits in episodic memory, i.e., the process through which details about previous experiences and events are stored, have been associated with various neurodegenerative and psychological disorders (e.g., anxiety disorders; for review see [19]). Examining whether such a relevant process can be reliably evaluated in web settings may have a positive impact on the detection of mnemonic dysfunctionalities and may facilitate the implementation of potential intervention programs. Thus, we examined the similarities between online and lab settings in the episodic memory performance. Both lab- and web-based experiments followed an identical protocol, in which neutral facial expressions differing in trustworthiness were encoded (i.e., free picture viewing procedure) and immediately retrieved (i.e., recognition memory procedures). Prior research found that untrustworthy faces are better remembered than trustworthy faces [20–23], a well-documented memory advantage that might serve an adaptive purpose to avoid potentially harmful social interactions [24]. If episodic memory processes can reliably be measured in web settings, the online and lab samples should show comparable levels of memory accuracy. In addition, the expected trustworthy effect (enhanced memory for untrustworthy faces) should be comparable in both samples. Furthermore, earlier studies showed mixed results on the interplay between episodic memory and anxiety disorders such as social anxiety (for review see [19, 25]. Thus, we also collected social anxiety scores from participants in the web sample as an efficient add-on to explore the influence of individual differences in social anxiety on memory performance for untrustworthy and trustworthy faces.

## Materials and methods

### Participants

A total of 33 students (30 female, 31 right-handed, *M*_*age*_ = 20.61 years, *SD*_*age*_ = 2.42 years) from the University of Greifswald participated in the lab study in exchange for course credits (for EEG results related to the encoding session see [26]) and 111 participants (87 female, 100 right-handed, *M*_*age*_ = 24.39 years, *SD*_*age*_ = 5.07 years) from the University of Potsdam completed the web study in exchange of course credits. All participants provided written-informed consent for a study procedure, which was approved by the ethics committee of the German Society for Psychology (DGPs) and the University of Potsdam and carried out in accordance with the Declaration of Helsinki. Before data analysis, the data was closely inspected to check that all participants executed the recognition memory task as instructed (e.g., by excluding participants who randomly guessed during their old/new judgement as indicated by Pr values equalling 0). After data inspection, no participants were excluded based on their overall Pr index, suggesting that participants in the unsupervised online environment took the study as seriously as in the supervised environment in the laboratory. However, seven participants from the web sample were excluded from the analyses (*N* = 3 were not students, *N* = 4 exceeded the overall completion time using the interquartile range criterion), leaving a final sample of 104 students (83 female, 94 right-handed, *M*_*age*_ = 24.08 years, *SD*_*age*_ = 4.91 years) in the web sample.

### Procedure

Both the lab study and the web study followed an identical protocol and consisted of two experimental sessions: an incidental encoding session and a recognition memory session, which took place immediately after the encoding session. The stimulus material consisted of 120 neutral Caucasian faces with direct gaze, which were previously evaluated as trustworthy (30 female, 30 male) and untrustworthy (30 female, 30 male) (same stimuli as in [22, 23]; [26] for details about stimulus construction and evaluation). The faces were converted into greyscales, position, and luminance and surrounded by an elliptic mask to minimize the influence of expression-irrelevant features on face perception during task (c.f., [22, 23, 26]).

During encoding, participants viewed a total of 60 neutral faces (30 trustworthy, 30 untrustworthy) presented in pseudorandom order. They were instructed to pay attention to the faces but neither informed that the faces differed in trustworthiness nor that a recognition test would follow (incidental encoding). Each trial began with a fixation cross presented for an interval that varied randomly between 1500 ms and 3000 ms, followed by a face presented once for 3000 ms. Directly after the free-viewing task, participants performed the recognition memory task. During the recognition memory task the previously seen neutral faces were presented intermixed with 60 new, i.e., not seen during encoding, neutral faces. Participants saw one face at a time (for 3000 ms) and were instructed to indicate (after the question old/new was presented) whether they had previously seen the stimulus during encoding (old face) or not (new face) by pressing the corresponding key on a keyboard (lab sample) or by clicking on the corresponding response field on the screen using their mouse or trackpad (web sample). The position of the response field on the screen was counterbalanced across participants, as were the response buttons on the keyboard in the lab environment. Following the old/new judgement, participants rated their memory confidence by pressing (lab sample) or clicking (web sample) on the corresponding percentage on a Likert scale ranging from 0% (i.e., not confident) to 100% (i.e., absolutely confident).

In the web sample, after the recognition session, participants completed the Liebowitz Social Anxiety Scale (LSAS; [27, 28]), which is a good proxy of the presence of social anxiety.

All stimuli in the lab setting were presented on a 27-in monitor (1920x1080 pixel) using Presentation® (Neurobehavioral Systems, Berkeley, CA), while participants were seated in a comfortable upholstered chair. The online experiment was implemented using the PsyToolkit platform [29, 30] and was automatically run on full-screen mode on modern web browsers (Mozilla Firefox, Microsoft Edge, and Google Chrome). The experiment was not compatible with Apple Safari browsers and did not run on a tablet or smartphone. Participants most frequently used a laptop with trackpad to complete the experiment (*N*_*laptop*,*trackpad*_ = 83, *N*_*laptop*,*mouse*_ = 10, *N*_*PC*_ = 11).

### Statistical analysis

To evaluate behavioral performance in both studies, the discrimination index Pr, *p*(*H*) − *p*(*FA*), and bias index Br, $\frac{p(FA)}{p(1-Pr)}$, were calculated overall and for trustworthy and untrustworthy faces, separately [31]. Higher Pr values are generally associated with better memory discrimination. Br values greater than 0.5 indicate a liberal response bias (i.e., bias to respond old), whereas lower values indicate a conservative response bias. We applied a two-sample Kolmogorov-Smirnov test to check for differences in Pr and Br distributions between the lab and the web samples [32]. To determine whether both experimental procedures differed in the recognition memory task and to replicate the trustworthy effects (i.e., enhanced memory for untrustworthy compared to trustworthy faces) in both samples, Pr and Br were further analyzed using a repeated-measures ANOVA with the within-subject factor Trustworthiness (trustworthy, untrustworthy) and the between-subject factor Sample (lab sample, web sample). For confidence ratings, a 3x2 ANOVA was performed using the factors Memory (hits, false alarms), Trustworthiness (trustworthy, untrustworthy), and Sample (lab sample, web sample). We further performed an exploratory analysis to evaluate potential gender differences in memory performance, independent of the task environment. To do so, all 24 male participants from both samples were pooled together with 24 randomly selected female participants from both samples that were matched in age. For Pr, we performed an ANOVA using this subsample (*N* = 48) with the within-subject factor Trustworthiness (trustworthy, untrustworthy) and the between-subject factor Gender (female, male). Correlational analysis was further performed to test trustworthiness-specific associations between memory performance and social anxiety scores, using Pearson’s correlation. The significance level for all analyses was set at *p* < 0.05. All statistical analyses were conducted using Rstudio [33].

## Results

### General memory effects

**Fig 1:**
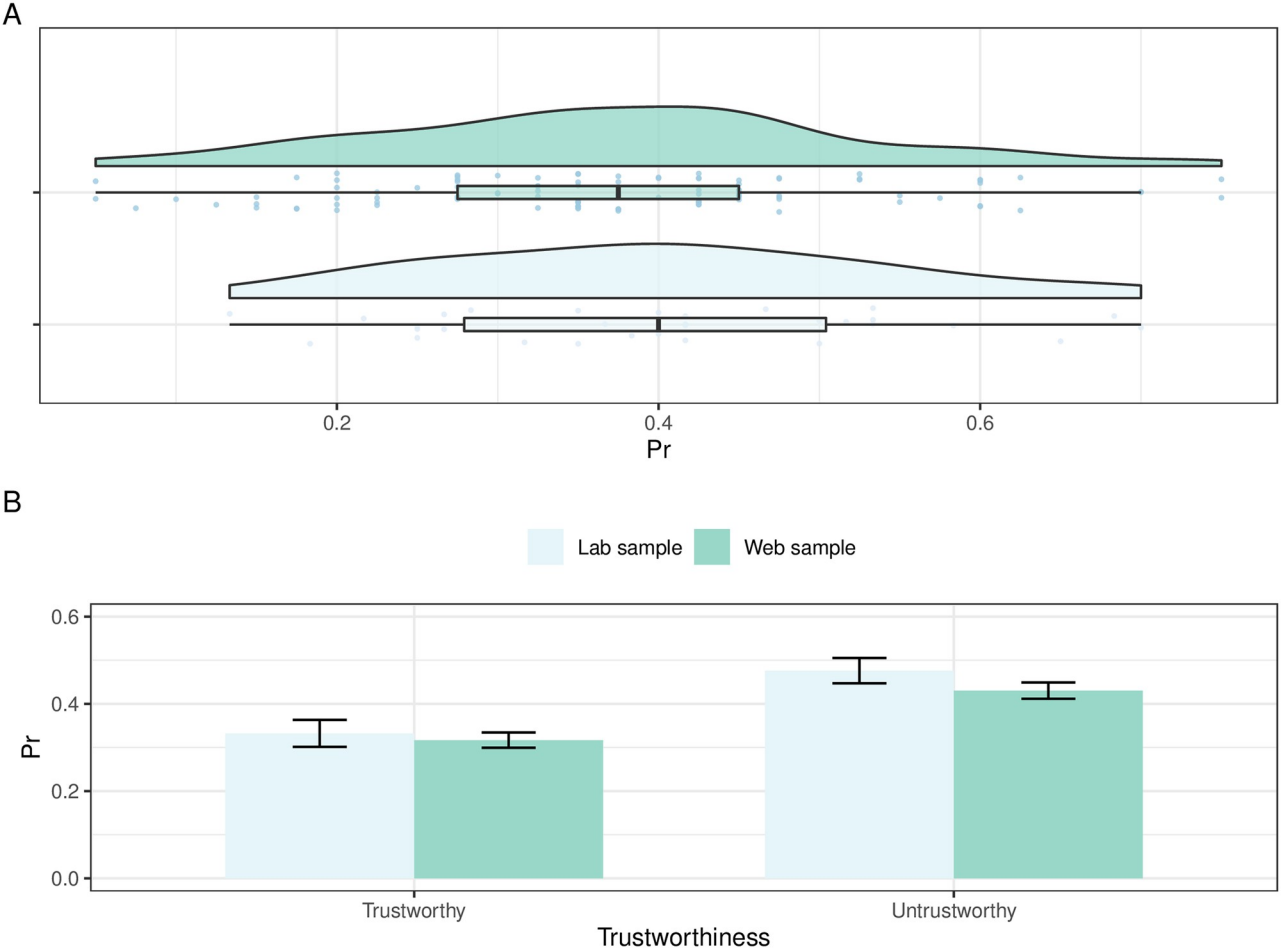
Raincloud plot and barplot. A: Visualization of raw data (rain drops) and distribution of the data (boxplot, half-side violin plot) of lab and web samples on discrimination index Pr. B: Barplot on discrimination index Pr showing the trustworthiness effects for the lab and web samples.

There was no significant difference in average memory performance between the lab and web sample, as indexed by Pr, *t*(134) = 1.02, *p* = 0.31 (*M*_*lab*_ = 0.40, *SD*_*lab*_ = 0.14; *M*_*web*_ = 0.37, *SD*_*web*_ = 0.15). Similarly, for Br, results showed no significant difference between samples, *t*(134) < 1 (*M*_*lab*_ = 0.45, *SD*_*lab*_ = 0.21; *M*_*web*_ = 0.42, *SD*_*web*_ = 0.17). Moreover, Pr and Br showed similar distributions across samples as indicated by the acceptance of the null-hypothesis of the two-sample Kolmogorov-Smirnov test: for Pr, *D*(134) = 0.15, *p* = 0.65, and for Br, *D*(134) = 0.16, *p* = 0.57 (see Fig 1).

When directly comparing all 24 male participants from both samples with 24 randomly selected female participants from both samples (matched in age), we did not find any sex-related differences in memory recognition, *F*(1, 90) = 0.39, *p* = 0.53, nor interactions of Gender and Trustworthiness, *F*(1, 90) = 0.11, *p* = 0.74.

### Effects of trustworthiness

Table 1 summarizes participants’ memory performance for trustworthy and untrustworthy faces in the lab and the web sample. When directly comparing the samples, for Pr, a main effect of Trustworthiness, *F*(1, 134) = 45.70, *p* < 0.001, $\eta_{p}^{2}=0.25$, indicated higher memory discrimination for untrustworthy, compared to trustworthy faces, irrespective of Sample (see Fig 1). Moreover, no Sample, *F*(1, 134) = 1.04, *p* = 0.31, $\eta_{p}^{2}=0.007$, or Sample x Trustworthiness effect, *F* < 1, were observed, suggesting that Sample did not have any specific effects on Pr (see Fig 1). For Br, results revealed a main effect of Trustworthiness, *F*(1, 134) = 22.00, *p* < 0.001, $\eta_{p}^{2}=0.14$, showing a more conservative response bias for untrustworthy than for trustworthy faces. A significant Trustworthiness x Sample interaction emerged, *F*(1, 134) = 6.87, *p* < 0.01, $\eta_{p}^{2}=0.05$, suggesting a more conservative response bias in the web sample for untrustworthy, compared to trustworthy faces.

**Table 1:** Descriptive statistics. Means (standard deviations) of behavioral indices for trustworthy and untrustworthy stimuli in direct comparison of the lab and of the web samples.

	Lab sample		Web sample	
	Trustworthy	Untrustworthy	Trustworthy	Untrustworthy
**Outcome rates**				
Hits (H)	.63(.18)	.70(.16)	.63(.17)	.64(.17)
False alarms (FA)	.30(.14)	.23(.13)	.32(.16)	.20(.12)
**Discrimination index**				
Pr	.33(.17)	.48(.16)	.32(.18)	.43(.19)
**Response bias index**				
Br	.46(.21)	.46(.27)	.46(.19)	.36(.19)
**Confidence ratings**				
Hits (H)	7.11(2.48)	7.47(2.57)	7.08(2.42)	7.54(2.48)
False alarms (FA)	5.31(2.39)	5.50(2.47)	5.74(2.28)	5.49(2.42)

For confidence ratings, a main effect of Performance was found, *F*(1, 262) = 14.24, *p* < 0.001, $\eta_{p}^{2}=0.63$, indicating higher confidence for hits compared to false alarms. Furthermore, a significant Trustworthiness x Performance interaction, *F*(1, 262) = 14.04, *p* < 0.001, $\eta_{p}^{2}=0.05$, revealed higher confidence for correctly retrieved old untrustworthy faces, compared to correctly retrieved old trustworthy faces, *t*(135) = −6.22, *p* < 0.01. Importantly, both, the Sample x Performance interaction (*F* < 1) and the Sample x Trustworthiness x Performance interaction, *F*(1, 262) = 2.26, *p* = 0.13, was not significant.

### Social anxiety and memory

Data on social anxiety were collected from participants in the web sample with the LSAS (*M*_*socialphobia*_ = 53.81, *SD*_*socialphobia*_ = 25.75, *M*_*avoidance*_ = 26.86, *SD*_*avoidance*_ = 13.92, *M*_*fear*_ = 26.95, *SD*_*fear*_ = 13.92). Correlational analysis did not reveal any significant relationship between the social anxiety scores and the Pr index either for trustworthy faces, *r*(102) = −0.04, *p* = 0.67, or for untrustworthy faces, *r*(102) = −0.08, *p* = 0.44. Similarly, no significant correlation was observed between the social anxiety scores and the Br index for any of the trustworthiness categories (−0.006 < *rs* < −0.02, *ps* > 0.83). Moreover, social anxiety scores did not correlate with participants’ overall confidence for trustworthy, r(102) = 0.05, p = 0.62, or for untrustworthy faces, *r*(102) = −0.04, *p* = 0.67. When considered separately, there were no significant correlations between the social anxiety scores and hits for any of the trustworthiness categories (−0.11 < *rs* < 0.04, *ps* > 0.28) (the same applies for false alarms, 0.08 < *rs* < 0.14, *ps* > 0.18).

Taken together, memory performance accuracy was comparable between the lab and web sample and showed comparable distributions, suggesting no effect of context on memory recognition memory. In addition, the trustworthy memory effect (i.e., enhanced memory performance for untrustworthy than for trustworthy faces) was similarly observed across samples. Significant differences only emerged for the Br index, suggesting that some disparities exist between both environmental settings.

## Discussion

The aim of the present study was to investigate the similarities between lab- and web-based settings in an episodic memory paradigm using trustworthy and untrustworthy faces. Our results showed that episodic memory performance was comparable (i.e., accuracy, distribution, confidence) across samples, suggesting that overall memory was not affected by the settings, in which the task took place.

We also observed a memory-enhancing effect for untrustworthy faces consistent with many previous studies [20–23]. This memory advantage may indicate that untrustworthy faces are highly relevant stimuli for organizing social behavior [34] because they might signal potentially dangerous or harmful encounters [24, 34]. As hypothesized, this trustworthy effect (i.e., enhanced memory for untrustworthy faces) typically found in a laboratory context was also replicated in the supposedly “uncontrolled” online environment suggesting that this effect is highly robust. Given the striking similarity in memory accuracy, our data, therefore, suggest that data quality was not affected when moving from laboratory to web-based testing, which is in line with several previous studies testing other psychological processes [14, 15, 17, 18, 35–39]. Our study further extends the validity of online experimental procedures to memory paradigms (e.g., [16, 40–43]).

Although memory performance accuracy was similar across settings, participants in the web sample showed a stronger conservative response bias for untrustworthy faces compared to trustworthy faces. The response bias has been described as the decision rule an individual is using when faced with uncertainty (i.e., on recognition memory tasks) [44]. It is theoretically independent of discriminability [31]. A shift in the response criterion has been previously observed when memory judgments become more difficult (e.g., by delay, see [45]) or when participants are under stress or threat (e.g., [46]). It is, at this point, speculative but the latter factor may have caused a change in response criterion (towards conservative) in the web sample given that the web-based study was conducted at a familiar non-stressed home, in which participants may show more cautious or controlled behavior than in the unfamiliar (lab-based), likely more stressful, environment. To address this response bias effect, however, future work should explore this possibility (i.e., under threat or stress conditions).

It is worth mentioning that both samples consisted predominantly of female participants which might have relevant implications in line with the evidence of gender differences in emotion processing and recognition (for review see [47]). Some previous studies observed that female compared to male participants are more reactive to emotional and stressful events as indicated by larger electrodermal activity and subjective ratings [48, 49]. However, experimental studies of sex differences in facial emotion recognition paradigms have reported contradictory findings (for sex-differentiated findings see [49–53]; but also see [54, 55]). Our exploratory analysis with a subsample of matched female and male participants, however, did not reveal any sex-related differences in recognition memory performance. Web-based experimental procedures provide an opportunity to shed further light on the disparity within the literature regarding biological sex differences (i.e., in a gender-matched sample directly testing for sex differences in memory for faces differing in trustworthiness) due to the facilitated recruitment of large and diverse samples of participants (e.g., in terms of gender) while keeping organizational issues and costs low (e.g., [5–7]).

Taking this advantage into consideration we were able to collect social anxiety scores of participants in the online experiment without much effort. This enabled us to explore the influence of individual differences in social anxiety on memory, which has been found in previous research to be enhanced, impaired or unaffected for facial expressions [19, 25]. In the present study, however, we found no indication of a significant relationship between social phobia scores and memory performance for facial expressions varying in trustworthiness which indicates no memory bias (e.g., [19]; c.f., [56] for role of social anxiety on trustworthiness judgments). It should be noted, however, that the social anxiety scores were obtained in the context of the Covid-19 pandemic, which has caused social withdrawal due to reasons other than social anxiety (i.e., social distancing, fear of infection). An emerging literature investigating the impact of social isolation and loneliness during the Covid-19 pandemic has shown increased depression and (social) anxiety symptoms from before the pandemic in samples of young adults [57–59]. Some evidence from our study may also point in that direction since social anxiety scores were higher compared to other representative samples (for American and British student sample see [60, 61]). Therefore, even when participants were instructed to fill in the questionnaire given usual habitual conditions it is not clear how individual responses were biased or affected by the pandemic, or possibly interfering with participants’ adherence to Covid-19 safety measures.

Web-based experimental procedures, however, provide an opportunity to accelerate, and even proceed (i.e., in the context of the Covid-19 pandemic) with empirical research and might lead the way in promoting transparency and reproducibility (i.e., access to the code alone would be adequate to completely replicate an experiment) in behavioral research [14]. Importantly, online testing might also be valuable for psychophysiological research [62, 63], particularly considering the current technological advantages. For instance, recent studies have shown that heart rate variability can be measured with smartphones (i.e., via video plethysmography by placing a finger on the camera lens of a smartphone [62]) or eye movements using webcams [63]. These measures have been only implemented in laboratory studies testing episodic memory for untrustworthy faces [23, 64], so far. Thus, the combination of behavioral and physiological measures in web-based and/or ambulatory settings may be a promising venue to investigate psychological processes in-situ (and in times of pandemic).

## Conclusion

This study tested the validity of a web-based episodic memory paradigm that included trustworthy and untrustworthy facial stimuli. We compared memory performance in a supervised laboratory setting with an unsupervised web setting and observed comparable memory effects. In both studies, we further found that untrustworthy faces were better remembered than trustworthy faces (replicating prior lab studies). Altogether, our findings suggest that online testing could be a promising tool for scientific research.

## References

[1] Peyton K., Huber G. A., & Coppock A. (2020). The generalizability of online experiments conducted during the COVID-19 pandemic.

[2] Sauter M., Draschkow D., & Mack W. (2020). Building, hosting and recruiting: A brief introduction to running behavioral experiments online. Brain sciences, 10(4), 251. 10.3390/brainsci10040251 PMC7226161 32344671

[3] Krantz J. H., & Dalal R. (2000). Validity of Web-based psychological research. In Psychological experiments on the Internet (pp. 35–60). Academic Press.

[4] Reips U. D. (2002). Standards for Internet-based experimenting. Experimental psychology, 49(4), 243. doi: 10.1026//1618-3169.49.4.241 12455331

[5] Birnbaum M. H. (2004). Human research and data collection via the Internet. Annu. Rev. Psychol., 55, 803–832. doi: 10.1146/annurev.psych.55.090902.141601 14744235

[6] Reips U. D. (2006). Web-based methods. In Eid M. & Diener E. (Eds.), Handbook of multimethod measurement in psychology (pp. 73–85). Washington, DC: American Psychological Association.

[7] Reips U. D. (2007). The methodology of Internet-based experiments. The Oxford handbook of Internet psychology, 373–390.

[8] Schmidt W. C. (2007). Technical considerations when implementing online research. The Oxford handbook of Internet psychology, 461–472.

[9] Berinsky A. J., Huber G. A., & Lenz G. S. (2012). Evaluating online labor markets for experimental research: Amazon. com’s Mechanical Turk. Political analysis, 20(3), 351–368. doi: 10.1093/pan/mpr057

[10] Buhrmester M., Kwang T., & Gosling S. D. (2016). Amazon’s Mechanical Turk: A new source of inexpensive, yet high-quality data?. 10.1177/1745691610393980 26162106

[11] Gosling S. D., Vazire S., Srivastava S., & John O. P. (2004). Should we trust web-based studies? A comparative analysis of six preconceptions about internet questionnaires. American psychologist, 59(2), 93. doi: 10.1037/0003-066X.59.2.93 14992636

[12] Linnman C., Carlbring P., Åhman Å., Andersson H., & Andersson G. (2006). The Stroop effect on the internet. Computers in human behavior, 22(3), 448–455. doi: 10.1016/j.chb.2004.09.010

[13] Germine L., Nakayama K., Duchaine B. C., Chabris C. F., Chatterjee G., & Wilmer J. B. (2012). Is the Web as good as the lab? Comparable performance from Web and lab in cognitive/perceptual experiments. Psychonomic bulletin & review, 19(5), 847–857. doi: 10.3758/s13423-012-0296-9 22829343

[14] Crump M. J., McDonnell J. V., & Gureckis T. M. (2013). Evaluating Amazon’s Mechanical Turk as a tool for experimental behavioral research. PloS one, 8(3), e57410. doi: 10.1371/journal.pone.0057410 23516406 PMC3596391

[15] Casler K., Bickel L., & Hackett E. (2013). Separate but equal? A comparison of participants and data gathered via Amazon’s MTurk, social media, and face-to-face behavioral testing. Computers in human behavior, 29(6), 2156–2160. doi: 10.1016/j.chb.2013.05.009

[16] Kleinberg B., & Verschuere B. (2015). Memory detection 2.0: The first web-based memory detection test. PloS one, 10(4), e0118715. doi: 10.1371/journal.pone.0118715 25874966 PMC4395266

[17] Bartneck C., Duenser A., Moltchanova E., & Zawieska K. (2015). Comparing the similarity of responses received from studies in Amazon’s Mechanical Turk to studies conducted online and with direct recruitment. PloS one, 10(4), e0121595. doi: 10.1371/journal.pone.0121595 25876027 PMC4397064

[18] Kim J., Gabriel U., & Gygax P. (2019). Testing the effectiveness of the Internet-based instrument PsyToolkit: A comparison between web-based (PsyToolkit) and lab-based (E-Prime 3.0) measurements of response choice and response time in a complex psycholinguistic task. PloS one, 14(9), e0221802. doi: 10.1371/journal.pone.0221802 31483826 PMC6726137

[19] Zlomuzica A., Dere D., Machulska A., Adolph D., Dere E., & Margraf J. (2014). Episodic memories in anxiety disorders: clinical implications. Frontiers in Behavioral Neuroscience, 8, 131. doi: 10.3389/fnbeh.2014.00131 24795583 PMC4005957

[20] Rule N. O., Slepian M. L., & Ambady N. (2012). A memory advantage for untrustworthy faces. Cognition, 125(2), 207–218. doi: 10.1016/j.cognition.2012.06.017 22874071

[21] Mattarozzi K., Todorov A., & Codispoti M. (2015). Memory for faces: the effect of facial appearance and the context in which the face is encountered. Psychological Research, 79(2), 308–317. doi: 10.1007/s00426-014-0554-8 24619533

[22] Weymar M., Ventura-Bort C., Wendt J., & Lischke A. (2019). Behavioral and neural evidence of enhanced long-term memory for untrustworthy faces. Scientific reports, 9(1), 1–8. doi: 10.1038/s41598-019-55705-7 31844252 PMC6915708

[23] Wendt J., Weymar M., Junge M., Hamm A. O., & Lischke A. (2019). Heartfelt memories: Cardiac vagal tone correlates with increased memory for untrustworthy faces. Emotion, 19(1), 178. doi: 10.1037/emo0000396 29553757

[24] Slepian M. L., Young S. G., Rule N. O., Weisbuch M., & Ambady N. (2012). Embodied impression formation: Social judgments and motor cues to approach and avoidance. Social Cognition, 30(2), 232–240. doi: 10.1521/soco.2012.30.2.232

[25] Staugaard S. R. (2010). Threatening faces and social anxiety: a literature review. Clinical psychology review, 30(6), 669–690. doi: 10.1016/j.cpr.2010.05.001 20554362

[26] Lischke A., Junge M., Hamm A. O., & Weymar M. (2018). Enhanced processing of untrustworthiness in natural faces with neutral expressions. Emotion, 18(2), 181. doi: 10.1037/emo0000318 28447825

[27] Liebowitz M. R. (1987). Social phobia. Modern problems of pharmacopsychiatry. 1987;22:141–73. doi: 10.1159/000414022 2885745

[28] Stangier U., Heidenreich T., & Scalarum C. I. P. (2005). Liebowitz Soziale Angst Skala [Liebowitz social anxiety scale]. Internationale Skalen für Psychiatrie, 299–307.

[29] Stoet G. (2010). PsyToolkit: A software package for programming psychological experiments using Linux. Behavior research methods, 42(4), 1096–1104. doi: 10.3758/BRM.42.4.1096 21139177

[30] Stoet G. (2017). PsyToolkit: A novel web-based method for running online questionnaires and reaction-time experiments. Teaching of Psychology, 44(1), 24–31. doi: 10.1177/0098628316677643

[31] Snodgrass J. G., & Corwin J. (1988). Pragmatics of measuring recognition memory: applications to dementia and amnesia. Journal of experimental psychology: General, 117(1), 34. doi: 10.1037/0096-3445.117.1.34 2966230

[32] Massey F. J. Jr (1951). The Kolmogorov-Smirnov test for goodness of fit. Journal of the American statistical Association, 46(253), 68–78. doi: 10.1080/01621459.1951.10500769

[33] RStudio Team (2021). RStudio: Integrated Development Environment for R. RStudio, PBC, Boston, MA URL http://www.rstudio.com/.

[34] Todorov A. (2008). Evaluating faces on trustworthiness: An extension of systems for recognition of emotions signaling approach/avoidance behaviors. Annals of the New York Academy of Sciences, 1124(1), 208–224. doi: 10.1196/annals.1440.012 18400932

[35] Dandurand F., Shultz T. R., & Onishi K. H. (2008). Comparing online and lab methods in a problem-solving experiment. Behavior research methods, 40(2), 428–434. doi: 10.3758/BRM.40.2.428 18522052

[36] Clifford S., & Jerit J. (2014). Is there a cost to convenience? An experimental comparison of data quality in laboratory and online studies. Journal of Experimental Political Science, 1(2), 120–131. doi: 10.1017/xps.2014.5

[37] Gould S. J., Cox A. L., Brumby D. P., & Wiseman S. (2015). Home is where the lab is: a comparison of online and lab data from a time-sensitive study of interruption. Human Computation, 2(1). doi: 10.15346/hc.v2i1.4

[38] Hilbig B. E. (2016). Reaction time effects in lab-versus Web-based research: Experimental evidence. Behavior Research Methods, 48(4), 1718–1724. doi: 10.3758/s13428-015-0678-9 26542972

[39] Armitage J., & Eerola T. (2020). Reaction time data in music cognition: Comparison of pilot data from lab, crowdsourced, and convenience Web samples. Frontiers in psychology, 10, 2883. doi: 10.3389/fpsyg.2019.02883 31969849 PMC6960264

[40] Verschuere B., & Kleinberg B. (2016). ID-check: Online Concealed Information Test reveals true identity. Journal of Forensic Sciences, 61, S237–S240. doi: 10.1111/1556-4029.12960 26390033

[41] Bentley S. V., Greenaway K. H., & Haslam S. A. (2017). An online paradigm for exploring the self-reference effect. PloS one, 12(5), e0176611. doi: 10.1371/journal.pone.0176611 28472160 PMC5417556

[42] Leding J. K. (2019). Intentional memory and online data collection: A test of the effects of animacy and threat on episodic memory. Journal of Cognitive Psychology, 31(1), 4–15. doi: 10.1080/20445911.2018.1564756

[43] Dorociak K. E., Mattek N., Lee J., Leese M. I., Bouranis N., Imtiaz D., et al. (2021). The Survey for Memory, Attention, and Reaction Time (SMART): Development and Validation of a Brief Web-Based Measure of Cognition for Older Adults. Gerontology, 1–13. doi: 10.1159/000514871 33827088 PMC8494835

[44] Huh T. J., Kramer J. H., Gazzaley A., & Delis D. C. (2006). Response bias and aging on a recognition memory task. Journal of the International Neuropsychological Society, 12(1), 1–7. doi: 10.1017/S1355617706060024 16433938

[45] Singer M., & Wixted J. T. (2006). Effect of delay on recognition decisions: Evidence for a criterion shift. Memory & Cognition, 34(1), 125–137. doi: 10.3758/BF03193392 16686112

[46] Qin S., Hermans E. J., Van Marle H. J., & Fernández G. (2012). Understanding low reliability of memories for neutral information encoded under stress: alterations in memory-related activation in the hippocampus and midbrain. Journal of Neuroscience, 32(12), 4032–4041. doi: 10.1523/JNEUROSCI.3101-11.2012 22442069 PMC6621211

[47] Dolcos F., Katsumi Y., Weymar M., Moore M., Tsukiura T., & Dolcos S. (2017). Emerging directions in emotional episodic memory. Frontiers in Psychology, 8, 1867. doi: 10.3389/fpsyg.2017.01867 29255432 PMC5723010

[48] Kring A. M., & Gordon A. H. (1998). Sex differences in emotion: expression, experience, and physiology. Journal of personality and social psychology, 74(3), 686. doi: 10.1037/0022-3514.74.3.686 9523412

[49] Kret M. E., & De Gelder B. (2012). A review on sex differences in processing emotional signals. Neuropsychologia, 50(7), 1211–1221. doi: 10.1016/j.neuropsychologia.2011.12.022 22245006

[50] McClure E. B. (2000). A meta-analytic review of sex differences in facial expression processing and their development in infants, children, and adolescents. Psychological bulletin, 126(3), 424. doi: 10.1037/0033-2909.126.3.424 10825784

[51] Hall J. A., & Matsumoto D. (2004). Gender differences in judgments of multiple emotions from facial expressions. Emotion, 4(2), 201. doi: 10.1037/1528-3542.4.2.201 15222856

[52] Montagne B., Kessels R. P., Frigerio E., de Haan E. H., & Perrett D. I. (2005). Sex differences in the perception of affective facial expressions: Do men really lack emotional sensitivity?. Cognitive processing, 6(2), 136–141. doi: 10.1007/s10339-005-0050-6 18219511

[53] Saylik R., Raman E., & Szameitat A. J. (2018). Sex differences in emotion recognition and working memory tasks. Frontiers in psychology, 9, 1072. doi: 10.3389/fpsyg.2018.01072 30008688 PMC6034094

[54] Grimshaw G. M., Bulman-Fleming M. B., & Ngo C. (2004). A signal-detection analysis of sex differences in the perception of emotional faces. Brain and cognition, 54(3), 248–250. doi: 10.1016/j.bandc.2004.02.029 15050785

[55] Derntl B., Finkelmeyer A., Eickhoff S., Kellermann T., Falkenberg D. I., Schneider F., et al. (2010). Multidimensional assessment of empathic abilities: neural correlates and gender differences. Psychoneuroendocrinology, 35(1), 67–82. doi: 10.1016/j.psyneuen.2009.10.006 19914001

[56] Cooper R., Doehrmann O., Fang A., Gerlach A. L., Hoijtink H. J., & Hofmann S. G. (2014). Relationship between social anxiety and perceived trustworthiness. Anxiety, Stress & Coping, 27(2), 190–201. doi: 10.1080/10615806.2013.834049 24041032

[57] Elmer T., Mepham K., & Stadtfeld C. (2020). Students under lockdown: Comparisons of students’ social networks and mental health before and during the COVID-19 crisis in Switzerland. Plos one, 15(7), e0236337. doi: 10.1371/journal.pone.0236337 32702065 PMC7377438

[58] Saraswathi I., Saikarthik J., Kumar K. S., Srinivasan K. M., Ardhanaari M., & Gunapriya R. (2020). Impact of COVID-19 outbreak on the mental health status of undergraduate medical students in a COVID-19 treating medical college: a prospective longitudinal study. PeerJ, 8, e10164. doi: 10.7717/peerj.10164 33088628 PMC7571415

[59] Hawes M. T., Szenczy A. K., Klein D. N., Hajcak G., & Nelson B. D. (2021). Increases in depression and anxiety symptoms in adolescents and young adults during the COVID-19 pandemic. Psychological Medicine, 1–9. doi: 10.1017/S0033291720005358 33436120 PMC7844180

[60] Wieser M. J., McTeague L. M., & Keil A. (2012). Competition effects of threatening faces in social anxiety. Emotion, 12(5), 1050. doi: 10.1037/a0027069 22390712 PMC3482481

[61] Russell G., & Shaw S. (2009). A study to investigate the prevalence of social anxiety in a sample of higher education students in the United Kingdom. Journal of Mental Health, 18(3), 198–206. doi: 10.1080/09638230802522494

[62] Peng R. C., Zhou X. L., Lin W. H., & Zhang Y. T. (2015). Extraction of heart rate variability from smartphone photoplethysmograms. Computational and mathematical methods in medicine, 2015. doi: 10.1155/2015/516826 25685174 PMC4309304

[63] Semmelmann K., & Weigelt S. (2018). Online webcam-based eye tracking in cognitive science: A first look. Behavior Research Methods, 50(2), 451–465. doi: 10.3758/s13428-017-0913-7 28593605

[64] Wang Q., Li Q., Chen K., & Fu G. (2015). Attentional Bias to Untrustworthy Faces: Evidence From Eye Tracking Data. Advances in Psychological Science, 23(9), 1508. doi: 10.3724/SP.J.1042.2015.01508

